# Supplementary material for: Curated and harmonised transcriptomics datasets of interstitial lung diseases
Source: Data Brief. 2025 Oct 14;63:112139. doi: 10.1016/j.dib.2025.112139 (PMC12581653; doi:10.1016/j.dib.2025.112139)
Supplement: Supplementary file 1 [file mmc1.zip › Supplementary_material/RNA-seq/fastQC reports/GSE184316/TRIMMED_SRR15926754_fastqc.html]

TRIMMED\_SRR15926754.fastq.gz FastQC Report 

FastQC Report

Sat 3 Dec 2022  
TRIMMED\_SRR15926754.fastq.gz

## Summary

- Basic Statistics
- Per base sequence quality
- Per sequence quality scores
- Per base sequence content
- Per sequence GC content
- Per base N content
- Sequence Length Distribution
- Sequence Duplication Levels
- Overrepresented sequences
- Adapter Content

## Basic Statistics

| Measure | Value |
| --- | --- |
| Filename | TRIMMED\_SRR15926754.fastq.gz |
| File type | Conventional base calls |
| Encoding | Sanger / Illumina 1.9 |
| Total Sequences | 5604926 |
| Sequences flagged as poor quality | 0 |
| Sequence length | 35-371 |
| %GC | 51 |

## Per base sequence quality

## Per sequence quality scores

## Per base sequence content

## Per sequence GC content

## Per base N content

## Sequence Length Distribution

## Sequence Duplication Levels

## Overrepresented sequences

| Sequence | Count | Percentage | Possible Source |
| --- | --- | --- | --- |
| CAGTGGGGCCACGAGCTGAGTGCGTCCTGTCACTCCACTCCCATGTCCCT | 116034 | 2.0702146647431205 | No Hit |
| GGCATCAAAAGTGATGGACTGCCCATTGCTGGAGAAGACCTTCTCTCCTA | 47805 | 0.8529104576938216 | No Hit |
| AGGCTACACACTGAGGACTCTGTTCCTCCCCTTTCCGCCTAGGGGAAAGT | 47091 | 0.8401716632833333 | No Hit |
| CAATCCACTTGATCCCAACTCATCTCTCATTTATTTCGGCTTCTTTTATT | 46690 | 0.8330172423329051 | No Hit |
| TACCACTGCCACCTTCTCCATCGGCTCCACTGGCCTCGTGGTGTATGACT | 35630 | 0.6356908191116172 | No Hit |
| TGATGACACCCACCGCAACTGTCTGTCTCATATCACGAACAGCAAAGCGA | 27125 | 0.4839492974572724 | No Hit |
| AGACATCAAATCCTGCAGACAAGGGGAGCCCTCAGTCTGCAGGGCTCCAT | 24324 | 0.43397539949679975 | No Hit |
| GTCCACGTCACACTTCATGATGGAGTTGAAGGTAGTTTCGTGGATGCCAC | 22083 | 0.3939927128386709 | No Hit |
| CTTCATGATGTAGCAGCAGGTGCCAGGGGCTGGCTTGTAGGCGATCAGCA | 21378 | 0.3814144914669703 | No Hit |
| GGCAATGAGCGGTTCCGCTGCCCTGAGGCACTCTTCCAGCCTTCCTTCCT | 16651 | 0.2970779632059371 | No Hit |
| TGCCATATCCACATGGAAAATCTCATCACCATCAAAGTCAAACATAAACT | 13337 | 0.23795140203456744 | No Hit |
| TGATATGGTTCCTGGCAAGCCCATGTGTGTTGAGAGCTTCTCAGACTATC | 13043 | 0.232706016100837 | No Hit |
| CAATGGGGTACTTCAGGGTCAGGATGCCACGCTTGCTCTGGGCCTCGTCG | 12361 | 0.22053814805048275 | No Hit |
| TTACAAAAGCAGAATAAAAGCGAAAAGAAATGAAAATGTTACACTACATT | 12055 | 0.21507866473170206 | No Hit |
| GTTCCATATCAGAGCTGTGATCTTGAGAGCCCTCTCCTTGGCTTTCCTGC | 11911 | 0.21250949611109943 | No Hit |
| GTCCACGTCACACTTCATGATGGAGTTGAAGGTGGTCTCGTGGATGCCGC | 10912 | 0.19468588880566845 | No Hit |
| AACAAGCACAAAGCCAAGAAAACCAACGATGCCAGATACTGAATTTCTAT | 10748 | 0.19175989120998208 | No Hit |
| CAGCATGTTCCCTCTCCTCATGAGATTGGTGAAGAAAGTATTTGGCAAAG | 10647 | 0.18995790488580938 | No Hit |
| GGCAATGAGCGGTTCCGGTGTCCGGAGGCGCTGTTCCAGCCTTCCTTCCT | 10552 | 0.18826296725416178 | No Hit |
| ACGTTTACCTGTCCATGTCTTACTACTTTGACCGCGATGATGTGGCTTTG | 10548 | 0.18819160145914504 | No Hit |
| CTCTCTTTCTGGCCTGGAGGCTATCCAGCGTACTCCAAAGATTCAGGTTT | 9466 | 0.1688871539071167 | No Hit |
| CGATCAGCACGGGCTCGTACTCTGCCACAAACTGATCACACTGCTTCTGG | 9308 | 0.16606820500395544 | No Hit |
| CCCTGGCTGCTACAGGAGAATAGCAGACAGGTATAGTTAAGAAACAACTT | 9301 | 0.16594331486267616 | No Hit |
| TCAGGAGACGCTCGTAGCCCTCGCGCTTCTCCTCGGCCAATTCGCGGAAG | 9231 | 0.1646944134498832 | No Hit |
| CTCCGCCGCCTCCGCCGCAGACGCCGCCGCGATGCGCTACGTCGCCTCCT | 9217 | 0.1644446331673246 | No Hit |
| AGACATCAAATCCTGCAGACAAGGGGAGCCCTCAGGTCTGCAGGGCTCCA | 9208 | 0.16428406012853694 | No Hit |
| GGGAAGCGGGGAATGTCTACGTGCGTATGCACGTGGCACTCTCTGCCCGA | 8830 | 0.15753999249945494 | No Hit |
| CTCCACCTACGGCGAGGGCGAGAGCGGCCCCATGGGCAACATCATGATCG | 8738 | 0.1558985792140699 | No Hit |
| TCAGGAGACGCTCGTAGCCCTCGCGCTTCTCCTCGGCCAGTTCGCGGAAG | 8734 | 0.15582721341905317 | No Hit |
| CAGCAGTTTACACCTGCTAGGGTGTTCAAAGGTCAGTGCTATAGAAATTC | 8411 | 0.15006442547145135 | No Hit |
| CGAAGCGGTTGTACTTGCGGATGTAGTGCAGATAGTCTCGGCGGATGACA | 7937 | 0.1416075787619676 | No Hit |
| TGTGGATGAATCTTGAACTGAGTTCCACTTGTAAACTTCTTGTTTCTTGT | 7832 | 0.13973422664277815 | No Hit |
| CAGTGGGCCACGAGCTGAGTGCGTCCTGTCACTCCACTCCCATGTCCCTT | 7593 | 0.1354701203905279 | No Hit |
| CGAGCCACAGCTCTGCCAGTACCCCAAGACTCAGCACTAGTCTGATGACC | 7568 | 0.13502408417167328 | No Hit |
| AATGGGCTGCCTGGAGCCCCTGGTGTCCCTGGAGAGCGTGGAGAGAAGGG | 7071 | 0.12615688414084325 | No Hit |
| CAATCCACTTGATCCCAACTCATCTCTCATTATTTCGGCTTCTTTTATTC | 6852 | 0.12224960686367671 | No Hit |
| GCCGACAAGACCAACGTCAAGGCCGCCTGGGGTAAGGTCGGCGCGCACGC | 6851 | 0.12223176541492252 | No Hit |
| CGTGAATACCAAGACCTGCTCAATGTTAAGATGGCCCTTGACATTGAGAT | 6407 | 0.1143101621680643 | No Hit |
| GCGTCTCTGTCTTCTTCAGTTTCGACTTATCGAATTTCTCGATCTCAGCC | 6387 | 0.1139533331929806 | No Hit |
| CAGGGTGAAGGCCCACCCGTGGAGGCCAGACCCAAAGCCCACGGTACCGA | 6262 | 0.11172315209870746 | No Hit |
| CCAAGATCTTCTTGATGTCCTTGGCGCTGGGGGAGGAGTTGCCCCCTAGG | 6137 | 0.10949297100443431 | No Hit |
| GGGAAGCGGGGAATGGTCTACGTGCGTATGCACGTGGCACTCTCTGCCCG | 5988 | 0.10683459514006072 | No Hit |
| AGCTGCTAAGTGCTGTGTTGTCGTTCCCCCTGCTTAAAATAAAGTTGTTT | 5917 | 0.10556785227851358 | No Hit |
| GGAGAAAACAGCACCAAGCAGGAGATCCTGGCTGCTCTTGAGAAAGGCTG | 5724 | 0.10212445266895584 | No Hit |
| GGGACCAAGGTCACCGTCCTAGGTCAGCCCAAGGCCAACCCCACTGTCAC | 5626 | 0.1003759906910457 | No Hit |

## Adapter Content

Produced by FastQC (version 0.11.7)
